# Shaping Quality in Cardiovascular Magnetic Resonance: A Comparative Study of Segmentation Approaches by Trainees and Experts

**DOI:** 10.1016/j.cjco.2025.05.003

**Published:** 2025-05-13

**Authors:** Jan Gröschel, Thomas Hadler, Leonhard Grassow, Hadil Saad, Darian Viezzer, Clemens Ammann, Leonora Zange, Florian von Knobelsdorff-Brenkenhoff, Edyta Blaszczyk, Jeanette Schulz-Menger

**Affiliations:** a Charité – Universitätsmedizin Berlin, corporate member of Freie Universität Berlin and Humboldt-Universität zu Berlin, ECRC Experimental and Clinical Research Center, Berlin, Germany; b Working Group on Cardiovascular Magnetic Resonance, Experimental and Clinical Research Center, a joint cooperation between Charité Medical Faculty and the Max-Delbrück Center for Molecular Medicine, Berlin, Germany; c DZHK (German Centre for Cardiovascular Research), partner site Berlin, Berlin, Germany; d Deutsches Herzzentrum der Charité – Medical Heart Center of Charité and German Heart Institute Berlin, Department of Cardiology, Angiology and Intensive Care Medicine, Berlin, Germany; e KIZ – Kardiologie im Zentrum, München, Germany

## Abstract

**Background:**

Cardiovascular magnetic resonance (CMR) is an established cardiovascular imaging (CVI) technique. Deficits in training limit the widespread use of CMR. This study analyzed the influence of CVI experience on segmentation, to define quality standards for teaching and supervision.

**Methods:**

Four CMR experts determined left ventricular (LV) and right ventricular (RV) gold-standard contours in end-systole (ES) and end-diastole (ED), by consensus. After a brief teaching session, readers independently performed segmentations. Readers were classified as beginners (no previous experience in CVI), intermediates (previous experience in CVI, but not in CMR), or experts (extensive experience in CVI including CMR). Results were compared, and the cause of deviation was analyzed, using metrics such as the Dice similarity coefficient (DSC).

**Results:**

A total of 46 readers (19 beginners, 21 intermediates, 6 experts) performed image analysis. Using the DSC, we found significant differences in endocardial LV ED contours (median [interquartile range]: beginners, 92.9% [91.9%-93.5%]); intermediates, 93.5% (93.0%-94.1%); experts, 93.9% (93.1%-94.3%); *P* = 0.043) and in myocardial contours (beginners, 79.0% (75.0%-80.9%); intermediates, 80.9% (78.0%-82.4%); experts, 85.0% (79.8%-86.5%); *p* = 0.001). Experts had higher DSC scores for the right ventricle (ES: beginners, 83.8% (81.3%-85.8%); intermediates, 81.7% (79.6%-85.6%); experts, 89.0% (86.6%-89.8%); *P* = 0.003; ED: beginners, 89.2% (88.1%-90.3%); intermediates, 88.6% (87.9%-89.2%); experts, 91.6% (89.8%-93.3%); *P* = 0.002). The disagreements were not traceable in absolute volume and function (*P* for all > 0.2). Sources of disagreement were related mainly to handling of basal slices.

**Conclusions:**

After a brief standardized teaching session, beginners and intermediates performed chamber quantification consistent with that of experts. Differences, especially in LV mass and RV segmentations, warrant continuous training, ideally accompanied by automatic methods for quality assurance.

## Graphical abstract

**Figure:**
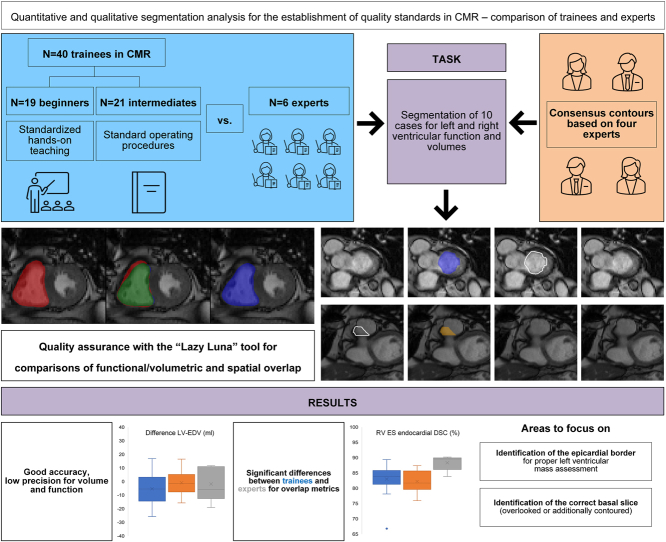

## Résumé

**Contexte:**

La résonance magnétique cardiovasculaire (RMCV) est une technique d'imagerie cardiovasculaire (ICV) bien établie. Les lacunes en matière de formation limitent sa diffusion à grande échelle. Cette étude a analysé l'influence de l'expérience en ICV sur la segmentation afin de définir des standards de qualité pour l'enseignement et la supervision.

**Méthodologie:**

Quatre experts en RMCV ont déterminé par consensus les contours de référence pour les ventricules gauche et droit (VG et VD) en fin de systole et de diastole (FS et FD). Après une brève formation, les opérateurs ont effectué les segmentations de manière indépendante. Les opérateurs ont été divisés en débutants (aucune expérience préalable en ICV), intermédiaires (expérience préalable en ICV, mais pas en RMCV) et experts (grande expérience en ICV, y compris en RMCV). Les résultats ont été comparés et les divergences analysées à l'aide de métriques telles que le coefficient de similarité de Dice (CSD).

**Résultats:**

Au total, 46 opérateurs (19 débutants, 21 intermédiaires, 6 experts) ont effectué l'analyse des images. En utilisant le CSD, nous avons trouvé des différences significatives dans les contours endocardiques du VG en FD (médiane (intervalle interquartile) : débutants 92,9 % (91,9 %-93,5 %); intermédiaires 93,5 % (93,0 %-94,1 %); experts 93,9 % (93,1 %-94,3 %); *p* = 0,043) et les contours myocardiques (débutants 79,0 % (75,0 %-80,9 %); intermédiaires 80,9 % (78,0 %-82,4 %); experts 85,0 % (79,8 %-86,5 %); *p* = 0,001). Les experts avaient des scores CSD plus élevés pour le VD (FS : débutants 83,8 % (81,3 %-85,8 %); intermédiaires 81,7 % (79,6 %-85,6 %); experts 89,0 % (86,6 %-89,8 %); *p* = 0,003; FD : débutants 89,2 % (88,1 %-90,3 %); intermédiaires 88,6 % (87,9 %-89,2 %); experts 91,6 % (89,8 %-93,3 %); *p* = 0,002). Les désaccords ne se retrouvaient pas au niveau des volumes absolus, ni de la fonction (*p* > 0,2 pour toutes les comparaisons). Les sources de divergences étaient principalement dues au traitement des coupes basales.

**Conclusion:**

Après une brève formation standardisée, les opérateurs débutants et intermédiaires ont effectué des mesures quantitatives des cavités cardiaques de manière cohérente avec les experts. Les différences, en particulier en ce qui concerne la masse du VG et les segmentations du VD, justifient une formation continue, idéalement accompagnée de méthodes automatiques d'assurance qualité.

Cardiovascular magnetic resonance (CMR) is recognized increasingly as a relevant imaging tool in the field of cardiology, as highlighted by the many CMR recommendations in various guidelines.^1^ Within a broad portfolio of sequences and post-processing methods, assessment of left and right ventricular (LV and RV, respectively) function remains one of the core utilizations of CMR.^2^ Quantitative assessment of biventricular volume and function has major impacts on diagnosis, prognosis, and therapy.^3^^,^^4^ For example, clinical decisions in regard to electrical device therapy are often based on the LV ejection fraction, or the size of the RV, as proposed in the current guidelines.^3^, ^4^, ^5^ Similarly, in research involving CMR, exact assessment of biventricular function is pivotal. Therefore, accurate and precise assessment of LV and RV volumes and function is crucial and is often the first step in the analysis of a CMR scan.

Training in CMR during a cardiology or radiology residency and fellowship is still often neglected or takes place shortly before board certification. Most curricula regarding CMR accreditation allow only board-certified physicians to obtain a certification.^6^^,^^7^ In addition, a discrepancy remains between high-volume and low-volume centres—for the latter especially, the evaluation of cardiomyopathies is a main indication.^8^ This indication, such as in hypertrophic or dilated cardiomyopathies, warrants an accurate evaluation of LV and RV function and volumes, as it also provides prognostication.

Previous studies in the field of CMR training have described an improvement in ventricular segmentation after providing a lecture-based and hands-on teaching course.^9^^,^^10^ Variance between beginners and experts remained after this initial teaching phase; these were especially pronounced in the assessment of LV mass and the determination of the most basal slice. Therefore, not only quantitative volume and function parameters need to be assessed during training, but also the segmentations themselves need to be checked and verified, to identify errors. The recent introduction of quality assurance tools, such as Lazy Luna (LL), provide clinically relevant values and spatial overlap metrics, such as the Dice similarity coefficient (DSC) and Hausdorff (HD) metrics.^11^ The DSC and the HD metrics are objective measures of overlap between 2 planes, or as in this study, 2 segmentations.^12^ Higher DSC values (in percent) signify a higher level of overlap and therefore better agreement between 2 readers. Given the simplicity of spatial overlap metrics, their use has been introduced recently in quality assurance and teaching in CMR.^11^^,^^13^ LL, a semiautomatic software, helps to detect divergences on the cohort level, as well as in inter-individual comparison.^11^^,^^13^

This study aims to provide, with the help of LL, the following: (i) insights into the precision and accuracy of beginners and intermediates regarding quantitative and qualitative segmentations in CMR after a standardized teaching session; and (ii) a framework for qualitative and quantitative parameters for CMR annotations in teaching courses.

## Methods and Materials

### Readers

Readers were divided into 3 groups as follows: beginners (no previous experience in cardiovascular imaging [CVI], including echocardiography and/or computed tomography or CMR); intermediates (previous experience in CVI, but not CMR); and experts (extensive experience in CVI, including CMR). Additional background information included field of work (physician, student, or study assistant). Physicians were further divided into residents and board-certified physicians, as well as by specialty (cardiology or radiology). Prior to image analysis, all readers except the experts received an introduction, including the current expert recommendations on segmentation,^2^ a standardized operating procedure on how to analyze LV and RV function, and a hands-on teaching session. The latter included an introduction to the analysis software, explanation of all features and necessary tools for contouring, and 2 case examples. In the case examples, emphasis was given to how to detect the basal slice, as well as the endocardial delineation of the blood pool. According to the current consensus statement,^2^ a basal slice for the left ventricle was defined as at least 50% of the blood pool being surrounded by myocardium, with the myocardium forming a “C.” Additional hints for identifying the proper basal slice were the following: tracking the movement of the left ventricle and the left atrium (the left atrium becomes smaller during the ventricular diastole); comparing the basal myocardial texture with a midventricular slice; and verifying that the myocardium is continuous, meaning that the slice is not angled and therefore includes part of the atrial wall. Use of artificial intelligence (AI)-based segmentations and tools was not permitted, so all contours were drawn from scratch. In addition, readers were tasked to erase any loaded workspace in the segmentation software before starting contouring, to prevent use or alteration of an existing workspace.

### Consensus cases and segmentations

Ten cases from clinical routine were randomly selected. All scans were acquired on a 1.5T Avanto^FIT^ scanner (Siemens Healthineers, Erlangen, Germany). Scans had to include a short axis (SAX) stack of balanced steady-state free precession cine images covering the entire left ventricle and right ventricle. According to local protocols, all SAX images were acquired after contrast-media application. Consensus contours (CCs) were determined via agreement by 4 CMR experts and were set as the gold standard. Endocardial contours were drawn in end-diastole (ED) and end-systole (ES) for the left and right ventricles. To delineate LV mass, additional epicardial LV contours were provided in ED. Papillary muscles were segmented separately in ED and ES and attributed to total LV mass. All CCs were based on the current expert recommendations.^2^ The software CVI^42^ (version 5.13.0, Circle Cardiovascular Imaging, Calgary, Canada) was used for image analysis.

### Quantitative segmentation analysis

The previously introduced LL tool^11^^,^^13^ was used to compare each individual reader and the CCs for quantitative function and volume parameters, as well as the DSC and the HD metrics. The DSC and the HD metrics were calculated in ED and ES for endocardial contours and in ED for LV myocardial contours (ie, the area between endocardial and epicardial contours). To minimize skewed outcomes, only slices with CCs were used for DSC calculation, thereby excluding slices with a mismatch (DSC of 0%) or without a contour (DSC of 100%) in the inter-reader–intra-slice analysis. Mismatches were present when the reader either ignored the slice for segmentation when a consensus contour was present, or conversely, inserted a contour when no consensus contour was present. A good agreement was defined as a DSC of ≥ 70%.^14^

In addition, readers were compared to the CC in terms of absolute function evaluation for the left ventricle and right ventricle, based on ejection fraction. Ejection fraction cutoffs for the left ventricle were as follows: > 55%, 55%-40%, 39%-30%, and < 30%. For the right ventricle, an ejection fraction of < 42% was used.

### Qualitative segmentation analysis

**Figure 1:**
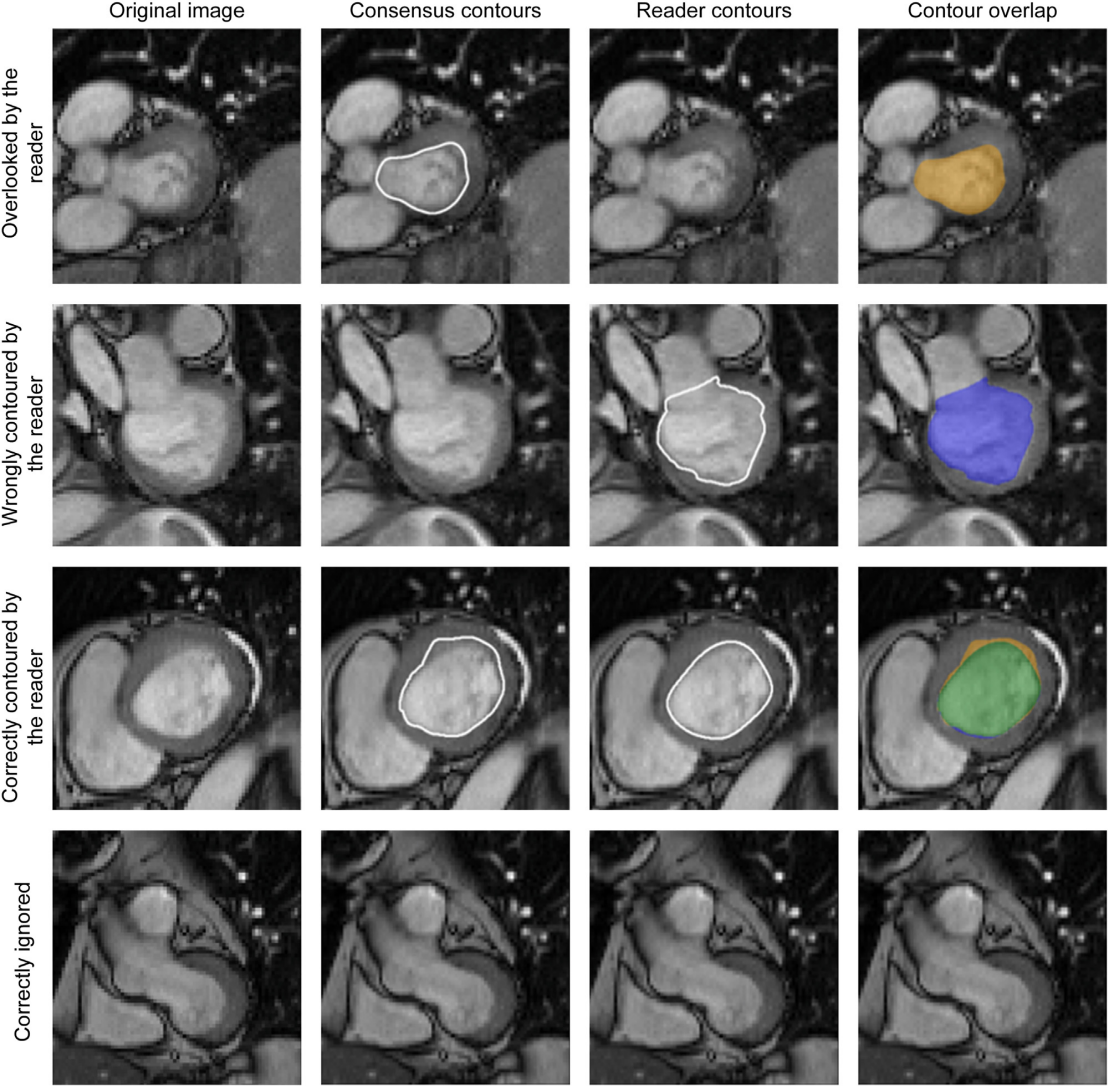
Examples for the segmentation analysis. Outputs of the Lazy Luna tool.^11^ The left images depict original images without contours. In the middle left are shown the consensus contours (CCs) of the experts; in the middle-right are the contours by the readers; and on the right are the overlap contours for the assessment of agreement (**green, overlap**; **yellow areas,** contoured by only the experts; **blue areas,** contoured by only the trainees).

The qualitative analysis of the segmentation by slice was divided into 4 categories: (i) segmentation ignored by the reader, vs present as a CC; (ii) segmentation by the reader without a CC; (iii) correctly contoured (ie, a CC and a reader contoured in the slice); and (iv) correctly ignored (ie, no CC and no reader contour in the slice; Fig. 1). In addition, we compared the chosen phase among the readers.

### Statistical analysis

To report accuracy and precision, the median and interquartile range (IQR) were reported for LV and RV functional and volumetric parameters, as well as LV mass. Intraclass correlation coefficients (ICCs) were calculated among all participants and for intragroup comparisons. Statistical comparisons over all 3 groups were carried out using analysis of variance and the Kruskal-Wallis test, as appropriate. Significant global tests were regarded as a strong trend and were followed with pairwise testing using the Student *t* test and the Mann-Whitney U test, as appropriate. We applied a Bonferroni correction to account for multiple testing. A *P* value < 0.05 was considered statistically significant. Statistical analysis was performed using SPSS, version 29.0.0 (SPSS, IBM, Armonk, NY) and R, version 4.4.3 (R Foundation, Vienna, Austria).

## Results

### Readers

In total, 46 readers provided segmentations (n = 19 beginners; n = 21 intermediates; n = 6 experts). For the final analysis, 46 complete LV datasets and 44 complete RV datasets (2 beginners did not provide RV segmentations) were available. The reader cohort consisted of n = 8 medical students, n = 14 residents in cardiology, n = 17 board-certified cardiologists, n = 6 board-certified radiologists, and n = 1 study assistant. The beginner group included n = 8 medical students, n = 5 residents in cardiology, n = 3 board-certified cardiologists, n = 2 board-certified radiologists, and n = 1 study assistant. The intermediate group consisted of n = 7 residents in cardiology, n = 11 board-certified cardiologists, and n = 3 board-certified radiologists. The expert group included n = 2 residents in cardiology, n = 3 board-certified cardiologists, and n = 1 board-certified radiologist.

### CCs

The 10 cases were acquired during clinical routine, consisting of 171 slices total. Nine of the patients were male (9 of 10, 90%). Basic characteristics (median [IQR]) were as follows: age, 68 years (54-75); weight, 82 kg (80-90); height, 178 cm (174-180); body surface area, 2.0 m^2^ (2.0-2.1); and heart rate, 64 beats per minute (55-70). The medians (IQRs) for volume and function parameters were as follows: LV ED volume, 147 mL (114-179); LV ES volume, 63 mL (52-81); LV stroke volume, 72 mL (64-108); LV ejection fraction, 58% (46%-64%); LV mass 110 g (103-114); RV ED volume, 152 mL (133-186); RV ES volume, 79 mL (68-92); RV stroke volume, 70 (56-95); RV ejection fraction, 48% (46%-51%). Three of the cases had an LV ejection fraction < 50%. Indications for scans were the detection of ischemia by stress perfusion in 8 of the cases, and 2 cases with suspicion of myocarditis. In 3 of 8 cases, the presence of ischemia was confirmed, whereas in the latter 2 cases, diagnosis of myocarditis was excluded. Four cases had visible scars on late–gadolinium enhancement images. Details for each case are given in Table 1.

**Table 1:** Individual volume and function parameters for the 10 training cases

Case	Gender	LV EDV, mL	LV ESV, mL	LV SV, mL	LV EF, %	LV mass, g	RV EDV, mL	RV ESV, mL	RV SV, mL	RV EF, %
1	M	65.1	29.4	35.7	54.8	108.6	90.4	47.0	42.2	46.6
2	M	178.9	103.0	75.8	42.4	110.7	157.7	76.5	80.0	50.8
3	M	158.5	92.6	66.0	41.6	143.8	133.2	66.6	61.2	45,99
4	M	170.0	63.4	115.5	64.5	113.1	192.5	94.3	99.7	51.8
5	M	109.8	60.6	49.2	44.8	63.8	114.6	62.1	55.4	48.3
6	M	100.8	31.1	69.7	69.2	100.9	132.7	70.3	59.7	445.0
7	F	207.4	72.5	134.8	65.0	107.4	222.8	105.7	129.5	58.1
8	M	134.9	49.6	85.3	63.2	115.0	167.6	82.1	79.1	47.2
9	M	125.2	62.2	63.1	50.4	97.7	146.4	83.6	47.1	32.1
10	M	209.1	83.2	126.0	60.2	113.9	270.6	136.9	136.3	50.4

EDV, end-diastolic volume; EF, ejection fraction; ESV, end-systolic volume; F, female; LV, left ventricular; M, male; RV, right ventricular; SV, stroke volume.

### Quantitative ventricular functional and volumetric parameters

**Figure 2:**
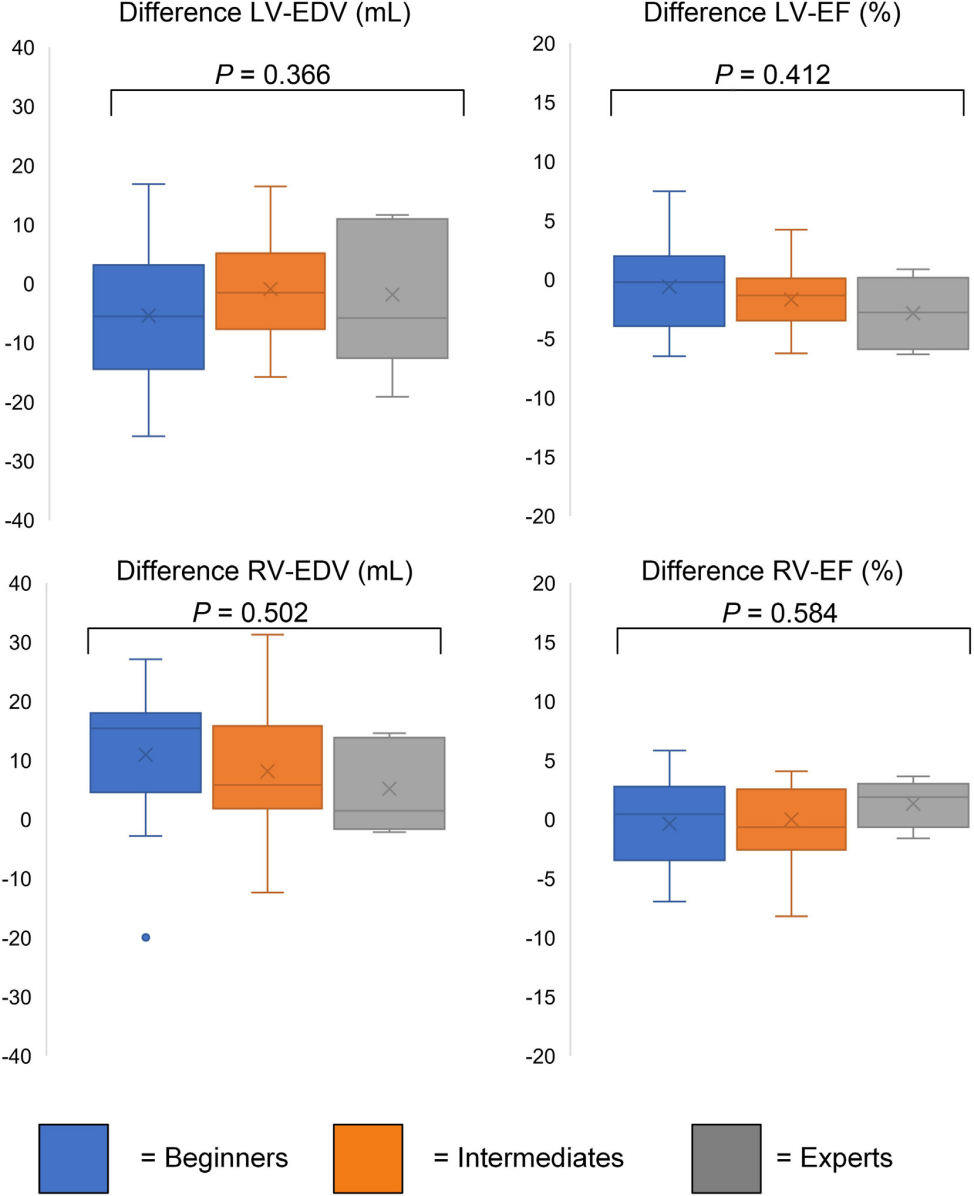
Boxplots for biventricular function and volumes. Boxplots representing the median (**solid line inside the box**), interquartile range (**box**), and 1.5∗interquartile range (**whiskers**) for beginners (**blue**), intermediates (**orange**), and experts (**grey**). Every value below or above 1.5∗interquartile range is marked as an outlier. All values are based on comparison between the “gold standard” consensus contours and the reader contours. EDV, end-diastolic volume; EF, ejection fraction; LV, left ventricular; RV, right ventricular.

No statistically significant differences occurred among the 3 groups in quantitative function or volume parameters (all *P* > 0.2; Table 2). Although the beginners and intermediates had a larger IQR, and therefore, lower precision than the experts, the median difference was close to zero for most parameters. Beginners had the highest median (IQR) difference for LV ED volumes (–5.6 mL [–14.4-3.2]), RV ED volumes (15.4 mL [4.6-18.0]) and RV ES volumes (7.9 mL [–0.4-14.4]; Table 2; Fig. 2).

**Table 2:** Results of left and right ventricular function analysis for beginners, intermediates, and experts

Parameter difference	Beginners (n = 19)	Intermediates (n = 21)	Experts (n = 6)	*P*
LV EDV, mL	–5.6 (–14.4–3.2)	–1.5 (–7.7–5.2)	–0.1 (–9.4–10.6)	0.366∗
LV EF, %	–0.2 (–3.9–2.0)	–1.3 (–3.5–0.1)	–1.7 (–5.7–0.8)	0.412∗
LV ESV, mL	–1.6 (–10.0–7.0)	0,6 (–3.1–3.5)	1.4 (–4.2–12.6)	0.220∗
LV SV, mL	–3.1 (–7.7–2.2)	–2.8 (–7.8–2.1)	–3.2 (–7.7–(–0.3))	0.870∗
LV mass, g	–3.1 (–7.7–2.2)	–2.8 (–7.8–2.1)	–2.2 (10.7–1.7)	0.753∗
RV EDV, mL	15.4 (4.6–18.0)	5.8 (1.8–15.8)	3.7 (–1.3–13.5)	0.502∗
RV EF, %	0.5 (–3.4–2.8)	–0.6 (–2.6–2.5)	1.1 (–0.6–2.7)	0.584†
RV ESV, mL	7.9 (–0.4–14.4)	5.4 (0.1–11.9)	2.8 (–0.6–4.9)	0.636∗
RV SV, mL	2.7 (0.5–10.7)	3.1 (–4.1–7.2)	1.4 (–1.3–9.8)	0.699†

EDV, end-diastolic volume; EF, ejection fraction; ESV, end-systolic volume; LV, left ventricular; RV, right ventricular; SV, stroke volume.

∗ *P* values with analysis of variance.

† *P* values with the Kruskal-Wallis test.

Analysis of the ICC for the entire cohort showed good agreement for all parameters except LV mass (ICC 0.39 [IQR 0.22-0.69]) and RV ejection fraction (0.35 [IQR 0.19-0.65]). As a subgroup, experts had the highest ICC (IQR) for all measured parameters, except LV ED volumes, for which the intermediate subgroup displayed a slightly higher ICC (intermediates 0.94 [0.87-0.98] vs experts 0.93 [0.75-0.98]; Table 3).

**Table 3:** Intraclass correlation coefficients for group comparisons

Group	LV EDV	LV EF	LV ESV	LV M	LV SV	RV EDV	RV EF	RV ESV	RV SV
All	0.92 (0.84–0.98)	0.78 (0.62–0.92)	0.87 (0.75–0.96)	0.39 (0.22–0.69)	0.90 (0.81–0.97)	0.91 (0.82–0.97)	0.35 (0.19–0.65)	0.71 (0.52–0.89)	0.82 (0.68–0.94)
Beginners	0.91 (0.80–0.97)	0.76 (0.5–0.92)	0.83 (0.67–0.95)	0.30 (0.14–0.61)	0.89 (0.79–0.97)	0.90 (0.78–0.97)	0.40 (0.21–0.70)	0.70 (0.48–0.89)	0.86 (0.73–0.95)
Intermediates	0.94 (0.87–0.98)	0.78 (0.62–0.92)	0.93 (0.86–0.98)	0.48 (0.26–0.77)	0.89 (0.78–0.97)	0.90 (0.80–0.97)	0.24 (0.11–0.54)	0.65 (0.45–0.87)	0.76 (0.59–0.92)
Experts	0.93 (0.75–0.98)	0.84 (0.61–0.95)	0.84 (0.55–0.95)	0.57 (0.21–0.85)	0.96 (0.89–0.99)	0.96 (0.89–0.99)	0.74 (0.53–0.91)	0.93 (0.85–00.98)	0.92 (0.82–0.98)

EDV, end-diastolic volume; EF, ejection fraction; ESV, end-systolic volume; LV, left ventricular; M, mass; RV, right ventricular; SV, stroke volume.

### Qualitative segmentation analysis

#### Spatial overlap metrics

**Figure 3:**
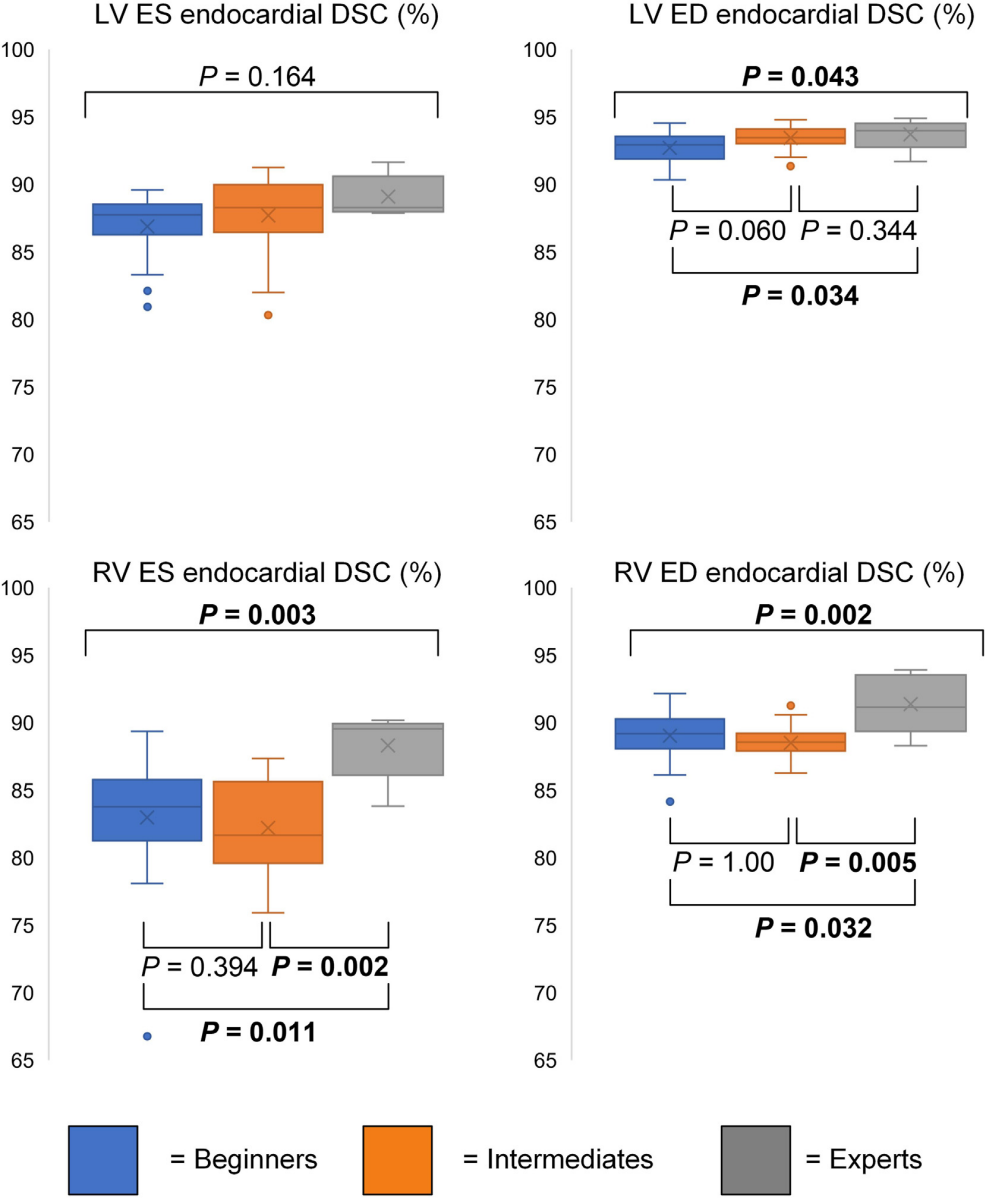
Boxplots for spatial overlap metrics. Boxplots representing the median (**solid line inside the box**), interquartile range (**box**) and 1.5∗interquartile range (**whiskers**) for beginners (**blue**), intermediates (**orange**), and experts (**grey**). Every value below or above 1.5∗interquartile range is marked as an outlier. All values are based on comparison between the consensus contours and the reader contours. DSC, Dice similarity coefficient; ED, end-diastole; ES, end-systole; LV, left ventricular; RV, right ventricular.

Overall good agreement between the CCs and the reader contours was noted, with the lowest DSC reported at 79.0% (75.0%-80.9%) for the beginners, for LV mass. Significant differences were detected for the ED LV endocardial DSC (*P* = 0.043), the ED LV myocardial DSC (*P* = 0.001), the ES RV endocardial DSC (*P* = 0.003), and the ED RV endocardial DSC (*P* = 0.002; Table 4). Except for the ES LV endocardial DSC, experts had the highest DSC. Significant differences between beginners and experts were detected for the ED LV endocardial DSC (*P* = 0.034), the ED LV myocardial DSC (*P* = 0.049), the ES RV endocardial DSC (*P* = 0.002), and the ED RV endocardial DSC (*P* = 0.032; Table 4). Intermediates and experts showed significant differences between only the ES RV endocardial DSC (*P* = 0.011) and the ED RV endocardial DSC (*p* = 0.005; Table 4; Fig. 3).

**Table 4:** Results of spatial overlap metrics for beginners (B), intermediates (I), and experts (E)

Parameter	B (n = 19)	I (n = 21)	E (n = 6)	*P*	B vs I	B vs E	I vs E
LV ES endocardial DSC, %	87.7 (86.3–88.5)	88.3 (86.4–90.0)	88.2 (88.0–90.1)	0.164∗	ns	**ns**	ns
LV ES endocardial HD, mm	4.3 (3.9–4.8)	4,4 (3,8–4,6)	4.4 (3.9–4.6)	0.774∗	ns	ns	ns
LV ED endocardial DSC, %	92.9 (91.9–93.5)	93.5 (93.0–94.1)	93.9 (93.1–94.3)	**0.043**∗	0.060	**0.034**	0.344
LV ED endocardial HD, mm	3.6 (3.4–4.0)	3.6 (3.2–3.8)	3.3 (3.1–3.9)	0.371†	ns	ns	ns
LV ED myocardial DSC, %	79.0 (75.0–80.9)	80.9 (78.0–82.4)	85.0 (79.8–86.5)	**0.001**†	**0.009**	**0.049**	0.365
LV ED myocardial HD, mm	4.2 (3.9–4.4)	4.1 (3.8–4.3)	3.7 (3.4–4.1)	0.111†	**ns**	**ns**	**ns**
RV ES endocardial DSC, %	83.8 (81.3–85.8)	81.7 (79.6–85.6)	89.0 (86.6–89.8)	**0.003**∗	0.394	**0.002**	**0.011**
RV ES endocardial HD, mm	8.8 (7.6–9.8)	9.0 (7.7–10.3)	5.5 (6.1–7.0)	**0.002**†	1.00	**0.02**	**0.008**
RV ED endocardial DSC, %	89.2 (88.1–90.3)	88.6 (87.9–89.2)	91.6 (89.8–93.3)	**0.002**†	1.00	**0.032**	**0.005**
RV ED endocardial HD, mm	8.1 (7.1–9)	8.5 (7.6–9.2)	6.4 (5.2–7.5)	**0.004**†	1.00	**0.018**	**0.010**

DSC, Dice similarity coefficient; ED, end-diastole; ES, end-systole; HD, Hausdorff unit; LV, left ventricular; ns, nonsignificant; RV, right ventricular.

∗ *P* values computed with the Kruskal-Wallis test.

† *P* values computed using analysis of variance; a *P* value < 0.05 was considered significant, *P* values in bold represent significant findings.

**Figure 4:**
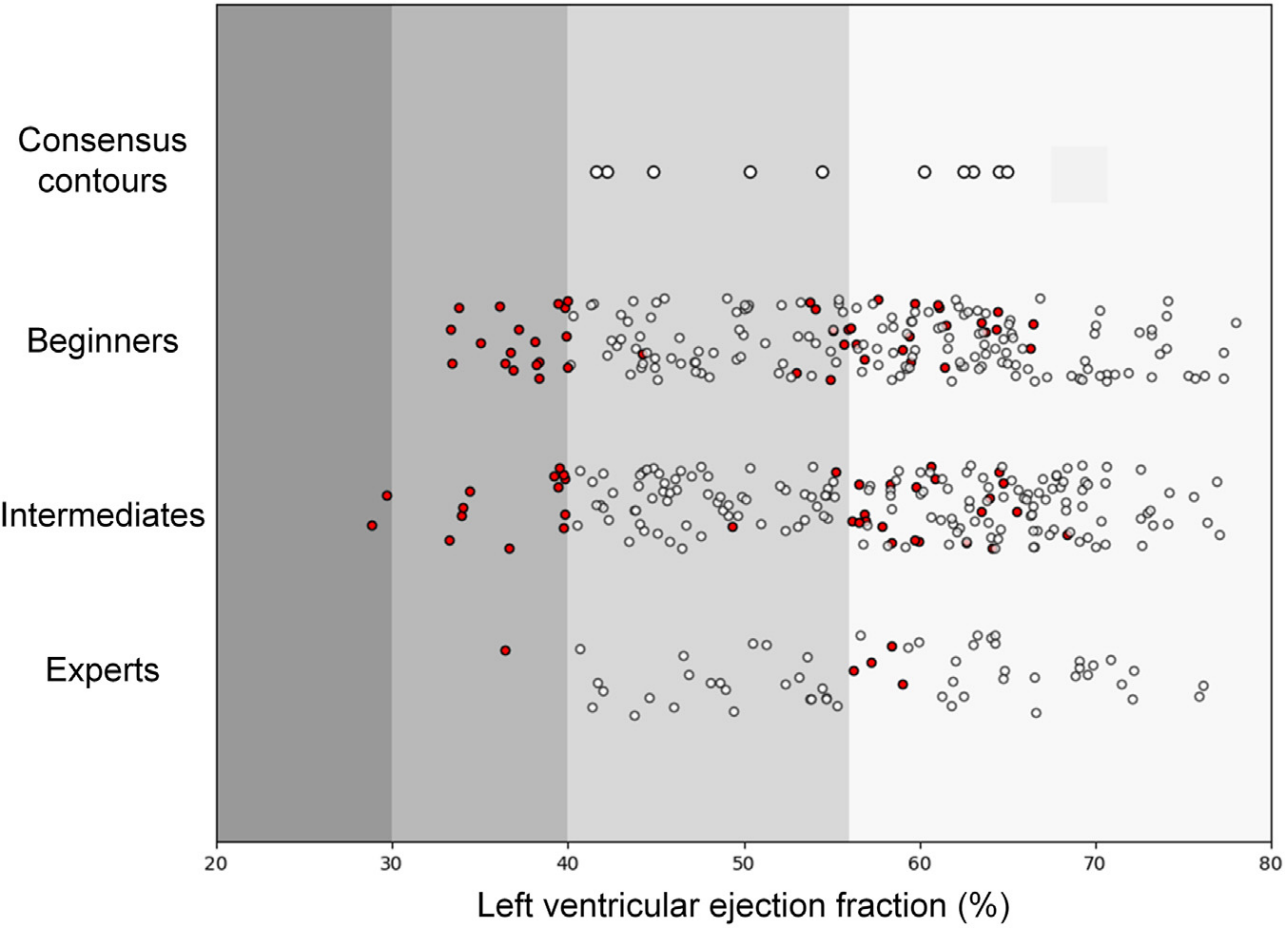
Left ventricular ejection fraction cut-offs. Shown are contoured cases with the consensus contours on the **upper row**. Each **circle** represents a case. **Red circles** signify misclassified cases.

**Figure 5:**
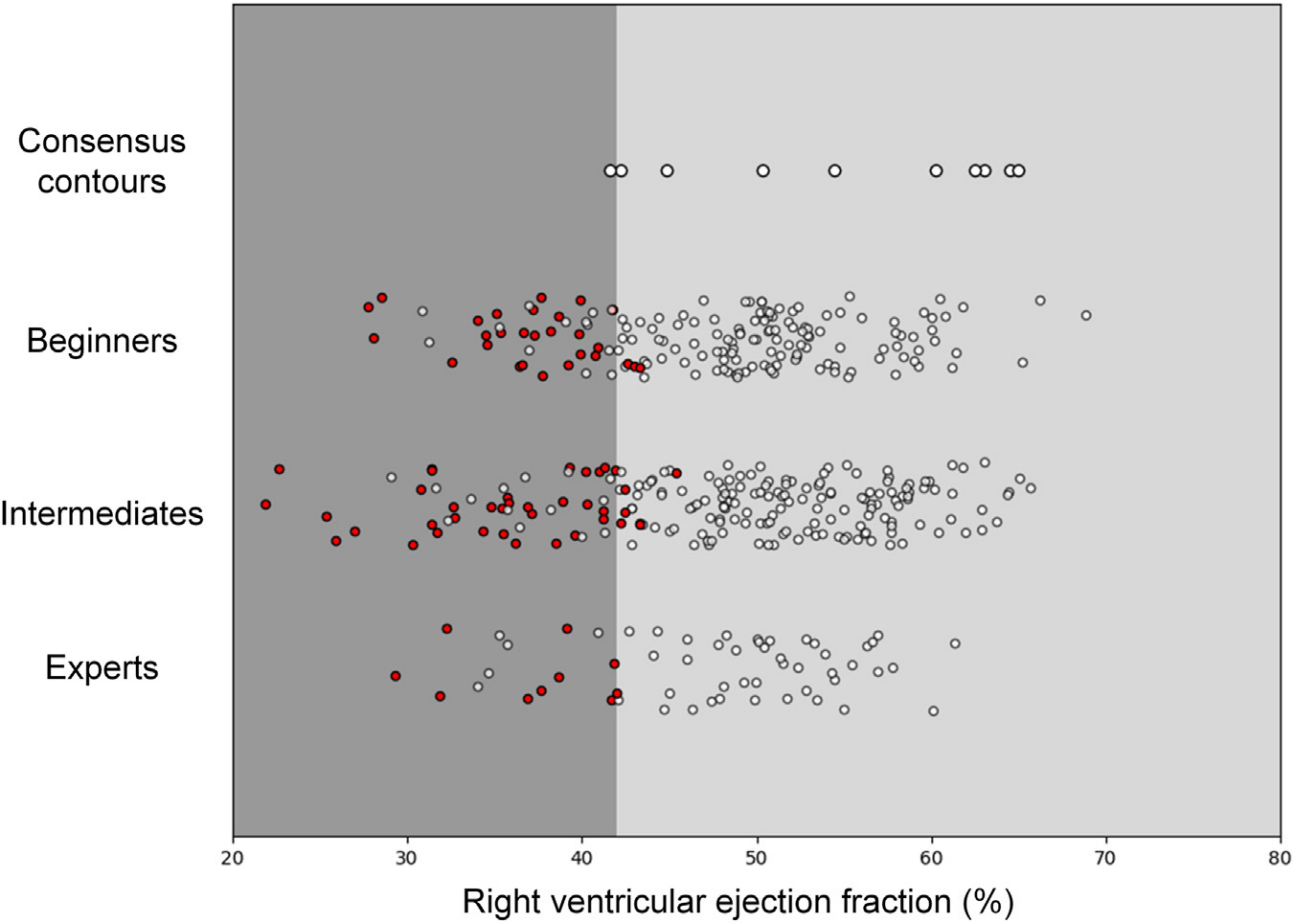
Right ventricular ejection fraction cut-off. Shown are contoured cases with the consensus contours on the **upper row**. Each **circle** represents a case. **Red circles** signify misclassified cases.

For the LV cutoffs for the ejection fraction, 44 cases were misclassified by the beginners, with 17 being classified incorrectly with an ejection fraction of < 40% (17 of 190; 9%). For the intermediates, 38 cases were misclassified, with 14 being classified incorrectly with an ejection fraction of < 40% (14 of 210; 7%), and 2 of these were misclassified with an ejection fraction of < 30%. The experts misclassified only 5 cases, and only 1 case was classified incorrectly with an ejection fraction of < 40% (1 of 60; 2%). For the RV ejection fraction, beginners misclassified 29 cases, 26 of them too low (26 of 170; 15%); intermediates misclassified 40 cases, 35 of them too low (35 of 210; 17%); and experts misclassified 11 cases, all of them too low (11 of 60; 18%; Figs. 4 and 5).

#### Qualitative segmentation analysis by slice

Given the difference in readers per group and whether the ED or the ES phase was analyzed, the maximum number of slices that could be contoured varied across the groups. Compared to the gold standard CCs, the beginners correctly segmented 3141 of 3249 slices (96.7%; = sum of correctly ignored and correctly contoured), intermediates 3203 of 3344 slices (95.8%), and experts 1012 of 1026 slices (98.6%) in terms of LVES endocardial contours. Similar results were achieved for the LV ED contours, with beginners contouring 3123 of 3249 (96.1%) correctly, intermediates 3162 of 3344 (94.6%), and experts 1014 of 1026 (98.8%). Slightly lower values were observed for the right ventricle regarding ES contours (beginners, 2608 of 2787 [93.6%]; intermediates, 3306 of 3520 [93.9%]; and experts, 485 of 513 [94.5%]) as well as ED contours (beginners, 2599 of 2787 [92.3%]; intermediates, 3271 of 3520 [92.9%], and experts, 984 of 1026 [95.9%]).

**Figure 6:**
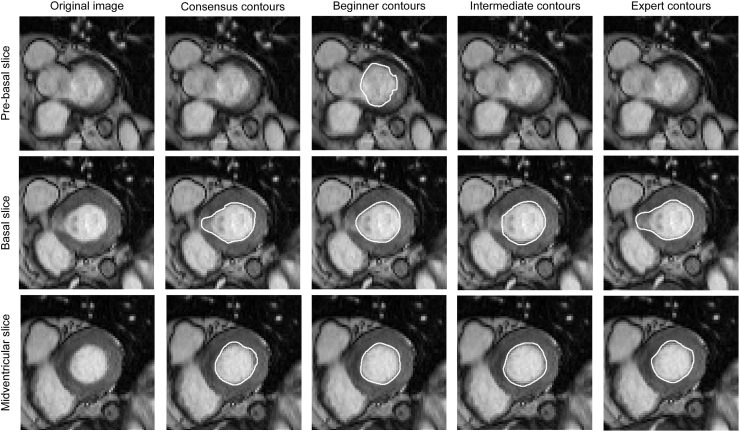
Examples for the segmentation analysis for basal slice choices. Outputs of Lazy Luna.^11^ The **left** images depict original images without contours. In the **column second from the left** are shown the consensus contours of the experts; in the **center column** are the beginner contours (notice the wrong choice of basal slice). The **column second from the right** are intermediate contours, and in the **right column** are the expert contours.

In the analysis regarding basal slice decisions, beginners wrongly contoured or overlooked slices in 54 of 190 cases (28.4%), intermediates in 72 of 210 cases (34.3%), and experts in 8 of 60 cases (13.3%) in the LV ES. For the LV ED basal slices, beginners wrongly contoured or overlooked slices in 72 of 190 cases (37.9%), intermediates in 82 of 210 cases (39.0%), and experts in 7 of 60 cases (11.7%) (Fig. 6). An overall higher rate of incorrect segmentations was noted in ES for the right ventricle (beginners, 98 of 190 [51.6%]; intermediates, 109 of 210 [51.9%]; and experts, 26 of 60 [43.3%]), as well as in ED (beginners, 104 of 190 [54.7%]; intermediates, 109 of 210 [51.9%]; and experts, 26 of 60 [43.3%]). Based on all readers for the LV ES and ED, basal slices were more often wrongly contoured (ES, 88 of 134 [65.7%]; ED, 116 of 161 [72.0%]). In contrast, for the RV basal segmentations, slices were more commonly overlooked (ES, 193 of 233 [82.8%]; ED, 234 of 239 [97.9%]). Regarding the phases chosen for the segmentation, beginners differed in comparison to the CCs by one phase in 9 of 19 cases (47%) for LV ED, 12 of 19 cases (63%) for LV ES, 8 of 17 cases (47%) for RV ED, and 11 of 17 cases (65%) for RV ES. Intermediates differed by one phase in 8 of 21 cases (38%) for LV ED, 8 of 21 cases (38%) for LV ES, 12 of 21 cases (57%) for RV ED, and 11 of 21 cases (52%) for RV ES. One expert differed in the LV ES phase decision, by one phase, and in the RV ED, by one phase. No reader differed for more than one phase from the CC.

## Discussion

A standardized approach to training can provide beginners and intermediates with the tools and knowledge to provide accurate assessment of biventricular function using CMR. This study did not show a difference in absolute quantification of LV and RV function and volumes across the different expertise levels. However, beginners and intermediates had a lower precision level in comparison to that of experts in CMR. This trend was also seen in classification according to the ejection fraction. Although the absolute differences were small, beginners and intermediates had lower overall spatial overlap metrics, in addition to segmentation errors, such as wrongly excluding or including a slice in the analysis. In contrast, for the left ventricle, the main issue was the additional contouring of slices; for the right ventricle, the basal slice was the most often overlooked. Lastly, segmentation of LV myocardium and right ventricle seemed to be even more challenging.

With the increasing availability of new sequences in CMR, analysis and post-processing with annotations and the final diagnosis can become a challenge for CMR readers. A central part of the quantitative analysis is the segmentation of cardiac structures, such as ventricle volumes, and the myocardium itself. Despite recommendations by experts, segmentations vary even among core laboratories.^15^ Proper evaluation requires structured teaching, as shown in previous studies comparing beginners before vs after training.^9^^,^^10^ Although the teaching course content and time differed among the studies, improved segmentations with lower absolute deviations from experts were noticed. An interesting point to note is that LV mass seemed to be the parameter, among all subgroups, with the largest variability, even after training. Accordingly, in this study, the DSC for myocardial contours differed both between beginners and intermediates and between beginners and experts. The DSC for the LV myocardium was, surprisingly, the lowest overall, compared to the DSC for the endocardial contours in ED and ES. Difficulties in performing LV mass segmentation might be the delineation of the border between the blood pool and the myocardium, as well as the definition of the epicardial contour. Epicardial fat tissue and chemical shift artifacts might contribute to difficulties delineating the epicardial border.^16^ Additionally, the use of contrast media prior to the SAX acquisition might pose a challenge for delineation.^16^

Unlike in the previous study, beginners and intermediates received a short teaching session with a focus on the major pitfalls, to eliminate uncertainties regarding the inclusion of papillary muscles and trabeculae in the LV mass. Another area of difficulty for beginners and intermediates was the RV assessment, as we noticed overall lower spatial overlap metrics in the ED and ES for the right ventricle. The main area of difficulty might be the choice of the basal slice. Beginners and intermediates most often overlooked basal slices. Even the expert group wrongly contoured 43.3% of slices compared to the CCs. The current society recommendation for the basal slice is inclusion of the area up to the pulmonary valve.^2^ In addition to choosing the right basal slice, choosing the correct phase seemed to be more challenging for the right ventricle. However, as in no case was the correct phase missed by more than one, the overall impact might be negligible.

Although we did not detect major differences between beginners and intermediates, experts clearly achieved higher DSC scores, as well as a higher ICC. This finding underlines the need for further training and supervision by experts. These differences are not limited to or only evident in CMR, but are present in other modalities of CVI, such as echocardiography^17^ and computed tomography.^18^

This study should also encourage beginners, especially residents, to start early with CMR, to obtain competency for their later professional career. This need is underlined by a recent survey indicating that the majority of CMR readers dedicate less than 25% of their time to CMR.^8^ The likelihood seems low that this time is spent on training. Only 36% of participants in the same survey reported receiving formal training.^8^ Efficient training is even more important in low-volume centres, which are increasing in number,^19^ and for women, who face additional challenges, such as financial and career disadvantages, as well as preexisting family duties.^20^

Although the introduction of AI in CMR has advanced the field, especially in the time-consuming annotation process, such as SAX analysis,^21^^,^^22^ the reader still needs competencies to detect and manually correct segmentation errors.^23^^,^^24^ These competencies are especially relevant when different AI models are applied.^25^ This fact underlines the need for standardized and time-efficient training, even in the era of progressively advancing AI-based segmentations, especially given that AI models struggle, as do trainees, in detection of the basal slices.

The discrepancy between the qualitative segmentation analysis based on spatial overlap metrics and the quantitative analysis regarding clinically relevant function and volume parameters underlines the need for proper quality assurance in CMR teaching. As hands-on courses with expert supervision are time-consuming, automated quality assurance procedures are warranted. The LL provides an automated comparison tool for quality assurance—for qualitative segmentation indices, such as spatial overlap metrics. and for quantitative indices relating to function and volume parameters. The tool also allows for integration of these variables into tolerance ranges, providing context for the description of accuracy and precision, as well as individual feedback.^11^^,^^13^ We therefore propose applying quality-assurance tools, such as LL, to increase accessibility to proper teaching resources and methods in CMR, especially for low-output centres, for those who are unable to attend hands-on sessions due to time and/or financial restrictions, and to avoid the environmental burden of travel vis a vis climate change. However, more research is warranted on the learning curve, to provide more evidence-based teaching. Potential formats include the LL tool as well as interactive learning formats.

Even though the group differences were subtle regarding the absolute values, we identified relevant differences in classification according to the ejection fraction for both ventricles. Although the absolute values matter, many treatment decisions are based solely on an ejection fraction below a certain cut-off—for example, implantation of devices or starting therapy.^3^ Therefore, cases with therapy-relevant results should be verified by a second expert reader.

### Limitations

Given the time-consuming segmentation task, only 10 cases and only 6 experts in CMR were involved in this study. Additionally, we enrolled neither a comparative group without a standardized introductory session nor a post-training comparison. No SAX stack was acquired prior to contrast-media application, providing further difficulties in the segmentation task for the readers. Only 3 cases had a reduced LV ejection fraction; therefore, no conclusions could be drawn in cases of patients with severely reduced ejection fraction and wall-motion abnormalities. Also, no cases with cardiomyopathies or congenital heart disease were included, reducing the generalizability of the results. Lastly, we did not include AI-derived contours in the analysis.

### Conclusion

After a short, standardized teaching course, beginners and intermediates reach a satisfactory level of accuracy for ventricular chamber quantification with CMR, albeit with a low level of precision. Differences, especially in determination of LV mass and RV segmentation, still exist. To overcome this limitation, training should follow predefined quality-assurance rules using dedicated tools and should be time-efficient, to enable residents and intermediates to develop competencies in CMR.
